# The effectiveness of radial shockwave therapy on myofascial pain syndrome: a two-armed, randomized double-blind placebo-controlled trial

**DOI:** 10.1186/s12891-025-08659-z

**Published:** 2025-04-24

**Authors:** Collins Ogbeivor, Huda AlMubarak, Tola Akomolafe, Hamad Alkahtani, Hussain AlMugizel, Inga Marin, Hala Aldosari, Nouf Aldhwayan, Gamal Mohamed, Khaled Alobthani

**Affiliations:** 1 Physical Rehabilitation Department, King Faisal Specialist Hospital and Research Centre, 11211 Riyadh, Saudi Arabia; 2 311 th Field Hospital Ft., US Army, Gillem, GA USA

**Keywords:** Myofascial pain syndrome, Shockwave, Sham (placebo), Extracorporeal wave therapy, Physiotherapy, Randomized controlled trial

## Abstract

**Background:**

Myofascial pain syndrome (MPS) is a common, costly, and often persistent musculoskeletal condition. Radial shockwave therapy (RSWT) is one of the most frequently used treatments for MPS. However, there is limited evidence to support its short-term effectiveness, primarily due to the poor methodological quality of the studies. This study aimed to determine the effectiveness of radial shockwave therapy, compared with placebo treatment, in patients with MPS in the neck and upper back.

**Method:**

A two-armed, randomized, double-blind, placebo-controlled trial was carried out in an outpatient physical rehabilitation department in a tertiary hospital. The sample comprised 70 adults aged 18 years or above with MPS. The intervention group received six treatment sessions. These consisted of RSWT: 1.5 bars (0.068 mJ/mm^2^), 2000 pulses, and a frequency of 15 Hz; and standard physical therapy stretches and exercises, including therapeutic home exercises. The control group received an identical treatment regime, except that they received a no-energy shock (nontherapeutic dose) of 0.3 bar (0.01 mJ/mm^2^). The outcome measures were the numeric pain score (NPS), neck disability index (NDI), pressure pain threshold (PPT) and SF-12 score at the 4-, 8- and 12-week follow-ups.

**Results:**

The study revealed a significant improvement (*p* < 0.05) in the NPS and PPT at the follow-up assessments (0–4, 0–8, and 0–12 weeks). The placebo group showed a significant difference in NDI scores at all intervals, whereas the shockwave group only showed significant improvement at 0–4 weeks. The shockwave group did not have significant changes in SF-12 scores, whereas the placebo group showed significant improvement in the SF physical score between 0–8 weeks (*p* = 0.01) and 0–12 weeks (*p* = 0.02). No statistically or clinically significant differences were observed between the placebo and shockwave groups across all outcomes at 4, 8, and 12 weeks.

**Conclusion:**

No significant differences were found between the placebo and shockwave groups at 4, 8, and 12 weeks. However, both groups showed statistically and clinically significant improvements in the NPS and PPT. Both groups showed improvements in the NPS and PPT scores; therefore, we recommend using radial RSWT as an adjunct to standard care, which includes therapeutic home exercises for individuals with MPS.

**Trial registration:**

The trial was prospectively registered on 19 April 2022 with https://clinicaltrials.gov/study/NCT05381987 and conducted according to Consolidated Standards of Reporting Trials (CONSORT) guidelines.

**Supplementary Information:**

The online version contains supplementary material available at 10.1186/s12891-025-08659-z.

## Introduction

Myofascial pain syndrome (MPS) is a prevalent and persistent musculoskeletal issue that affects a large portion of the general population, with a lifetime prevalence ranging from 30 to 93% among individuals experiencing musculoskeletal pain [1–4]. In the United States, it is estimated to affect approximately 9 million people, with similar figures found in Canada [5]. Although rates of MPS vary between males and females, MPS is more common in adults older than 60 years. The prognosis varies widely, with many patients reporting persistent pain even after many years [5, 6]. MPS can affect various body parts and is commonly believed to be triggered by factors such as poor posture, prolonged use of display screen equipment, and jobs requiring static neck and shoulder positions. Over time, these factors can lead to reduced joint and myofascial mobility, impact activities of daily living, increase work absenteeism, and decrease overall quality of life [7, 8]. Furthermore, MPS may lead to social withdrawal and contribute to mood disorders such as depression and anxiety [7, 8]. Considering its significant impact on work efficiency and quality of life, timely, personalized, and effective treatment approaches are crucial for achieving optimal outcomes.

The causes of MPS and its underlying factors remain unclear; as a result, the pathophysiology of the condition is not fully understood [9]. However, MPS is characterized by the presence of myofascial trigger points (MTrPs), which are hyperirritable, palpable nodules located within skeletal muscle fibres [1, 10]. There is currently no gold standard or consensus on the treatment modalities for MPS, and the effectiveness of these interventions remains debatable. Additionally, treatment responses can vary significantly from patient to patient and are influenced by both extrinsic and intrinsic factors. Patients with MPS may experience localized pain, muscle tenderness, a palpable intramuscular taut band, a local twitch response, referred pain, muscle spasm, and sleep disturbance [11–14]. The assessment of MPS typically involves a combination of subjective and objective evaluations. This includes palpating the affected area, checking the response, and measuring pressure pain thresholds via a digital algometer [15].

Several interventions are available for MPS, such as deep frictional massage, acupuncture, kinesiotaping, local anaesthetic injections, low-level laser therapy, dry needling, and ultrasound therapy [10, 11, 16, 17]. However, there is currently no gold standard or consensus on the most effective treatment modalities, and regardless of modality, the effectiveness of these interventions remains debatable. Furthermore, treatment responses can vary significantly from patient to patient and are influenced by various extrinsic and intrinsic factors. Lifestyle factors [18], perceived disability [19], individuals'perceptions of treatment benefits and barriers [20], and poor compliance with treatment protocols can affect treatment outcomes. Identifying the most effective and efficient intervention for MPS is crucial for preventing symptom persistence, improving clinical outcomes and patient experiences, and ultimately reducing healthcare costs.

Radial shockwave therapy (RSWT) is a widely used treatment for MPS, recommended by the National Institute for Clinical Excellence (NICE, 2022) [21] for certain tendinopathy conditions and FDA-approved. This non-invasive method provides a mechanical stimulus to musculoskeletal tissue pain, offering lower peak pressure while maximizing energy delivery to the skin, allowing waves to propagate outward without a distinct focal point [22, 23]. RSWT uses a device to transmit controlled, short-duration acoustic shockwaves through the skin to the affected area [21]. RSWTs are generated by a pneumatic device that propels a projectile into a transmitter, transforming kinetic energy into shockwaves that disperse radially [22, 23]. Unlike focused shockwaves, radial shockwaves cover a wider area, making them effective for superficial treatments [22, 23]. RSWT is characterized by minimal negative side effects and utilizes low- to medium-energy pulses, with typical penetration depths ranging from 3 to 4 cm (1.2 to 1.6 inches). The physical effects of Extracorporeal Shock Wave Therapy (ESWT) are closely related to energy per unit area (EFD, mJ/mm^2^) or the maximal positive pressure (bar), which serves as a measure of the ESWT dosage [24]. For reference, a standard measure of ESWT at 1 bar is approximately 0.045 mJ/mm^2^ [24]. Therapeutic doses of ESWT can range from low levels of < 1.78 bar (< 0.08 mJ/mm^2^) to high levels of up to 13.3 bar (0.63 mJ/mm^2^) as noted by Rompe et al. [25]. Each treatment session typically administers between 1,000 and 2,500 shocks, while the total number of sessions generally varies from three to seven.

Specific therapeutic doses of RSWT can be found in the published protocol of Ogbeivor et al. [26].

The precise physiological mechanisms of ESWT for musculoskeletal conditions remain not fully understood; however, shockwaves are believed to facilitate tissue healing and modify pain signaling through various mechanical and cellular effects. These include mechanical stimulation and increased local blood flow; enhanced microfunctional and microstructural changes; and the release of substances such as substance P, prostaglandin E2, and tumor growth factor (TGF-β) [27–29]. Additionally, RSWT has a transient analgesic effect on afferent nerves and contributes to the breakdown of calcific deposits, which can potentially lead to tissue repair and regeneration [27–29].

Although the pathophysiological mechanisms explaining the effectiveness of shockwave therapy for MPS in the neck are not well defined [30], several authors [31–34] have endorsed its use. A recent systematic review and meta-analysis of randomized clinical trials revealed that, compared with control and ultrasound therapy, the RSWT is effective in relieving pain and improving functionality in patients with myofascial pain syndrome regardless of location. However, the authors did not specify exactly what the control was and it is not exactly clear from the review how many participants with MPS in the neck and upper back improved significantly compared to the control. In addition to these deficits, Avendaño-López and colleagues reported that the protocols and parameters for applying shockwave therapy were heterogeneous across eighteen studies that used the RSWT as a treatment choice [9]. The meta-analyses published by Yoo et al. [35] and Jun et al. [28] included five and 11 studies, respectively. While Yoo et al. [35] compared the effectiveness of extracorporeal shockwave therapy (ESWT) in the treatment of MPS in the neck and shoulder with that of other treatments, Jun et al. [22] investigated the effect of ESWT exclusively on the trapezius muscle. This study provides minimal evidence supporting the use of the RSWT for the short-term alleviation of neck pain in MPS patients. The authors emphasized the necessity for large-scale, high-quality, placebo-controlled trials in this field, given the small sample sizes and poor methodological quality of the existing studies. Moreover, in previous studies [30, 33, 36], the effectiveness of shockwave therapy was compared, but these studies were not purely sham (placebo)-controlled trials. A suitable sham (placebo) should be biologically inactive and psychologically credible, meaning that it should be indistinguishable from the real intervention to the patient [37]. In this study, a sham shockwave produces sound, making it psychologically credible and similar to a real shockwave [26]. However, it is physiologically different from the real shockwave because it is biologically inactive (no therapeutic dose). Therefore, considering the painful nature of the treatment in both groups, the sham may not entirely eliminate a placebo effect.

### Objective and hypotheses

The primary objective of this randomized controlled trial (RCT) was to compare the short- and medium-term effects of radial shockwave therapy in reducing pain and disability and improving the function of patients with MPS in the neck and upper back. The hypothesis is that, compared with placebo, radial shockwave therapy results in better outcomes in terms of pain and function.

### Research question

Is radial shockwave therapy more effective at improving MPS in the neck and upper back than sham therapy (placebo)?

## Methods

### Study design

The study protocol was published previously [26] and followed the CONSORT recommendations. This was a two-armed, randomized, double-blind (patient- and assessor-blind) placebo-controlled trial. Ethical approval for the study was granted on 10 April 2022 by the Research Ethics Committee of King Faisal Specialist Hospital and Research Centre (KFSHRC) with study number (RAC# 2221047). The trial was prospectively registered on 19 April 2022 with https://clinicaltrials.gov/study/NCT05381987. This study was conducted according to the Consolidated Standards of Reporting Trials (CONSORT) guidelines for clinical trials (See attachment in the additional file).

### Setting and participants

The study took place in the outpatient physical rehabilitation department of KFSHRC, Riyadh, where participants were recruited.

Eligible participants were adults (≥ 19 years) who had neck and/or upper back pain localized to the lateral or posterior neck and/or upper back, palpable tenderness in the lateral or posterior neck and/or upper back, single or multiple points in the lateral or posterior neck and/or upper back and who were able to provide informed consent. The exclusion criteria were as follows: participants (< 19 years) with a history of malignancy; lung tissue; haemophilia; anticoagulant therapy; visible tissue damage (skin petechiae and microvasculature disruption); metal implants; implanted cardiac stents and heart valves; infection; rheumatic, respiratory, and cardiovascular diseases; psychopathy; disorders of the vestibular and visual systems; and neck or shoulder surgery within a year. Other criteria include a recent history of steroid injections for myofascial trigger points, pregnancy, a diagnosis of fibromyalgia, cervical radiculopathy or myelopathy, inability and unwillingness to continue study and failure to provide consent.

Participants were recruited through referrals from general practitioners and specialists such as orthopedic consultants and physical therapists during their initial physical therapy appointments. Those who provided written informed consent and met the eligibility criteria after being screened by the assessing and treating physical therapist (PT) were enrolled in the study. A PT assessed them in person to confirm that they had a diagnosis of MPS in the neck and/or upper back and would benefit from radial shockwave therapy. The PT was used to determine the presence or absence of active myofascial trigger points in the neck and upper back using the eligibility criteria defined by previous authors [14, 15]. Participants who declined to participate in the study received standard PT treatment. We recruited 70 participants for this study, with an average of 110 new participants per month.

### Randomization and blinding

**Fig. 1:**
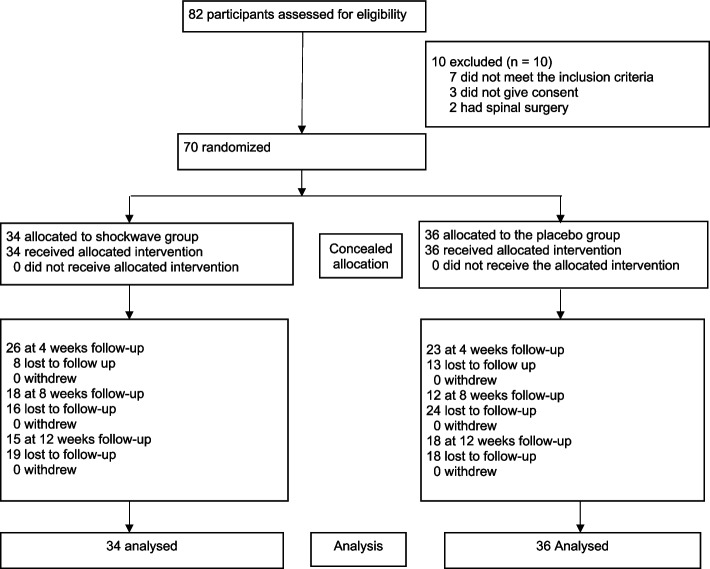
Flowchart showing the movements of patients throughout the trial

After completing the baseline questionnaire, the participants were randomly assigned to the intervention (shock wave therapy) or sham (placebo) group at a 1:1 allocation ratio. The randomization was based on a computer-generated random sequence with a permuted block size of 4 and concealed random allocation via sealed opaque envelopes by administrative staff who were not involved in screening, randomization, or data collection. The randomization sequence was performed via sequentially numbered, otherwise identical, sealed envelopes with a written code A or B designating the intervention (radial shockwave therapy) or control (sham (placebo), respectively. On the basis of the notification of the randomization result by the administrative staff, the treating PT assigned the participants to the treatment allocations via label A or B on the sealed envelope. Details of the participant flow through the study are shown in Fig. 1. The physical therapists delivered either of the treatments but were not involved in the data entry. All participant baseline and outcome measures were collected and entered into the SPSS spreadsheet by a research team member who was masked to the group allocation. Data analysis was conducted by a senior biostatistician and epidemiologist who were blinded to the baseline measurements and group allocations. All the authors agreed upon the interpretation of the results before unmasking.

### Interventions

#### Intervention (Experimental) group

The participants in this group received a total of 6 sessions, with a one-week interval, of radial shockwave therapy via a (Storz Medical) device. The device was set to the following parameters: 0.57 mJ/mm2 (1.5 bar), delivering low-energy pulses at a frequency of 15 Hz, and using the D20 transmitter (Ø 20 mm) headpiece.

#### Control (Sham [placebo]) group

The control group received an identical treatment regimen except that they received a no-energy shock of 0.3 bar [0.01 mJ/mm^2^] (an ineffective [nontherapeutic]) level of RSWT [25]. The participants were blinded to their treatment by hearing only the sound from the shockwave machine.

For both groups, the therapists ensured that the treatments were carried out via aseptic techniques and that the participants'skin was intact. To confirm the participants'myofascial triggers, a twitching response was induced via a digital algometer by the treating physical therapist and then the RSWT was applied directly to the identified trigger or painful point (trapezium/periscapular area).

The initial consultation lasted 45 min and included a thorough history taking, physical examination, and educational guidance. It also encompassed shockwave treatment, standard physical therapy stretches and exercises, along with therapeutic home exercises. The 30-min follow-up session focused on shockwave treatment, additional stretches and exercises, and assessing the participants'adherence to their home therapeutic exercises. The stretches and exercises included Quadruped Cat/Camel, wall arm slide, wide arm push-ups (including wall push-ups, modified floor push-ups, and floor push-ups), neck rotation stretch, and horizontal shoulder adduction stretch. Each exercise was performed for 10–15 repetitions, 2 sets, and 3–4 times per week. These exercises target the lower cervical, scapular, and upper thoracic muscles. Experienced physical therapists skilled in shockwave therapy administered these treatment sessions and assessed participants before treatment. All the participants received the same exercises and were able to carry them out regardless of their pain, disability, or age.

All the participants received verbal aftercare and post treatment information, including advice to continue their normal daily activities. Only one participant reported increased pain after the intervention, and no other serious adverse effects were reported in this study. The participants who experienced additional pain after treatment were managed according to normal clinical procedures by the head of the department's research committee.

#### Baseline characteristics

The baseline characteristics recorded included age, sex, duration of symptoms, current treatment with analgesics and current treatment with NSAIDs. They also included the initial numeric pain scale (NPS), neck disability index (NDI), pressure‒pain threshold (PPT) and short-form health survey (SF- 12) scores.

#### Outcomes and follow-ups

The primary measure of effectiveness was the NPS, a single 11-point scale ranging from 0 for"no pain"to 10 for"worst imaginable pain". It has been used to assess the intensity of pain in adults with musculoskeletal disorders, such as MPS [38]. The scale was considered reliable, responsive, and valid according to Hawker [38]. A decrease of 2 points or 30% in the NPS represents the minimal clinically important difference (MCID), as defined by Childs et al. [39] and Farrar et al. [40]. The NDI was a primary outcome that was used as a self-rated disability score for participants with MPS. It includes ten domains and is scored on a 0–50 scale, with 0 being the best and 50 being the worst [41]. The score can also be reported as a percentage (0–100%). The minimum detectable change is 5 points, and the MCID is in the range of 3.5–5.0 points [41, 42].

The secondary outcomes included the PPT and SF- 12 scores. PPT was assessed via a digital algometer, and pain scores were measured via digital palpation. The algometer had a circular flat tip with a 1.0 cm2 surface area and was slowly applied vertically to the skin over the trigger point(s) until the participant felt pain. The pressure was applied at a rate of 1 kg/cm2. The participants were instructed to indicate when they felt pain by saying"yes"to the treating physiotherapist. The measurements were repeated three times at 40-s intervals, and the average value was recorded. A mean difference of 0.94 kg/cm2 in PPT is considered clinically meaningful [43]. The physical and emotional aspects of quality of life status (SF- 12) were measured via a 12-item questionnaire. Scores ranged from 0 to 100, with higher scores indicating better health. The SF- 12 is widely used for musculoskeletal patients and has high validity and reliability [44]. A Saudi version with high reliability and validity scores is available [45]. A minimal improvement of 20% in the SF- 36 score was defined as the MCID by Lauche et al. [46].

The participants were assessed three times during the study period: at 0 (baseline), 4, 8, and 12 weeks. Follow-up assessments were conducted by a staff member who was not involved in the patient’s treatment and was blinded to the baseline measurement and group allocation at 4, 8 and 12 weeks.

#### Loss to follow-up

The study lasted for 12 weeks; therefore, at 4 weeks, the rate of loss to follow-up was reviewed to ensure that this did not affect the findings of the study. Patients who were lost to follow-up were included in the analysis on the basis of intention to treat (ITT) using last observation carried forward See Fig. 1.

#### Data and treatment fidelity

The treating physiotherapists were skilled, trained and experienced in the management of MPS RSWT. The treatments they provided were routinely evaluated by the principal investigator (CO) to ensure the procedural integrity of the study. Some of the treatment sessions from both groups of the study were observed and documented, and feedback was provided to the treating physiotherapists. Standardized training on the study procedure was provided to the treating physiotherapists to facilitate the successful delivery of both treatments. The administrative staff involved in the study received training on the study protocol. The radial shockwave machines for this study passed their normal checks to ensure that they were properly calibrated and working well. A Data Monitoring Committee did not find anything untoward. All the data were accessible only to the research team.

### Statistical analysis

#### Sample size calculations

Sample size calculations were based on works by Aktürk et al. [47] and Gur et al. [33]. We estimated the minimal clinically important difference (MCID) to be a change in the NPS of 2 points at 90% power, with a statistical significance level of 5% and a standard deviation of 4.35 points. Using these figures, a sample of 96 participants was estimated as having sufficient power. To account for a 20% rate of loss to follow-up, 120 participants were needed for the study. However, 70 individuals participated in the study.

#### Plan of analysis

All analyses were conducted on an intention-to-treat (ITT) basis. Descriptive statistics, including the means (± SD) for age, sex, and duration of symptoms, were reported for participants'baseline characteristics and outcomes at 4, 8, and 12 weeks. Normality was assessed via the Kolmogorov‒Smirnov test because the sample size was 70. The chi-square test was used to compare the distributions of categorical variables. Within-group differences were analysed via a paired sample t test, whereas between-group differences were assessed via an independent sample t test. A regression model was used to evaluate the impact of participants' baseline characteristics, such as age, sex, and symptom duration. The significance level was set at *p* ≤ 0.05 with a 95% confidence interval to detect a minimal clinically important difference of 2 points between the groups receiving shockwave therapy and those receiving sham (placebo) treatment. Statistical analysis was carried out via IBM SPSS Statistics version 20 (SPSS Inc., Chicago, IL, USA).

## Results

Participants were recruited into the study between April 2022 and July 2023. A total of 102 participants with MPS were approached for entry into the trial, but 32 were excluded. Among those excluded from the study, 12 did not have a diagnosis of MPS, 14 did not fulfil the injection eligibility criteria, and 6 refused to participate in the study. Therefore, 70 participants with a diagnosis of MPS who fulfilled the study's eligibility criteria were recruited: 34 (48.6%) in the shockwave group and 36 (51.4%) in the placebo group (See Table 1). Twenty (29%) participants were lost to follow-up at 4 weeks**.** In these cases, participants could not be reached by telephone. However, the data were analysed on the basis of intention-to-treat analysis via the last observation. One participant discontinued treatment at 4 weeks because of increased pain.

**Table 1:** Numbers of participants recruited

**Participants**	**Shockwave group**	**Placebo group**	**Totals**
**Male**	27	25	52
**Female**	7	11	18
**Totals**	34	36	70

The baseline characteristics for both the shockwave and placebo groups were similar for age, sex, duration of symptoms, current treatment analgesia and current treatment NSAIDs. Both groups had similar NPS, NDI, PPT and SF- 12 scores. Table 2 shows the baseline characteristics of the study sample. There was no statistically significant difference between the means of the two groups at baseline, which meant that the randomization process was adequate.

**Table 2:** Baseline characteristics according to group

**Characters**	**Shockwave group**	**Placebo group**	***P* value**
**Age in Years, mean (SD)**	44.79 (10.54)	45.61 (12.61)	0.77*
**Age groups in years**			**0.367**
18–40	14 (41.2%)	13 (36.1)	
41–65	20 (58.8%)	21 (58.3)	
> 65	0 (0.0%)	2 (5.6)	
**Gender**			0.34**
Male	27 (51.92)	25 (48.08)	
Female	7 (38.89)	11 (61.11)	
**Manual occupation**			0.42**
Yes	11 (42.31)	15 (57.69)	
No	23 (52.27)	21 (47.73)	
**Current Pain Medication**			0.86*
Yes		12 (50)	
No		24 (52.17)	
**Duration of symptoms in weeks, mean (SD)**	46.97 (91.46)	50.83 (77.65)	0.84*
**Duration group of symptoms in weeks**			**0.174**
0–12	24 (70.6%)	18 (50.0%)	
13–26	4 (11.8%)	5 (13.9%)	
** > 26**	6 (17.6%)	13 (36.1%)	

^*^ = t test

^**^ = chi-square

### Primary outcomes

Table 3 shows the results of an independent t test of the NPS, PPT, NDI, and SF12 scores between the two treatments at baseline, week 4, week 8, and week 12. There was no statistically significant difference between the groups in these measures at baseline, week 4, week 8, or week 12 (*p* > 0.5).

**Table 3:** Results of NPS, PPT, NDI, and SF12 scores between the two treatments at baseline, week 4, week 8 and week 12 using independent t test

	Placebo group	Shockwave group	Mean difference (95% confidence interval of difference)	*p* value
	Mean (SD)	Mean (SD)		
**Patient Reported Outcome Measure**				
**NPS**				
Week 0	7.1 (1.4)	6.9 (1.9)	0.20 (− 0.58, 0.99)	0.609
Week 4	4.7 (2.6)	4.9 (2.9)	− 0.27 (− 1.60, 1.05)	0.681
Week 8	4.5 (2.6)	4.7 (2.8)	− 0.22 (− 1.50, 1.06)	0.735
Week 12	3.5 (2.6)	4.6 (2.5)	− 1.10 (− 2.33, 0.13)	0.078
**PPT**				
Week 0	2.8 (1.7)	2.4 (1.4)	0.34 (− 0.40,1.07)	0.364
Weeks 4	3.3 (2.1)	3.0 (1.3)	0.24 (− 0.63, 1.1)	0.588
Week 8	3.3 (2.1)	3.0 (1.4)	0.32 (− 0.53, 1.17)	0.457
Week 12	3.4 (2.1)	3.1 (1.5)	0.314 (− 0.57, 1.2)	0.479
**NDI**				
Week 0	31.1(15.9)	29.6 (15.5)	1.41 (− 6.09, 8.91)	0.709
Week 4	24.6 (16.8)	24.1 (14.0)	0.50 (− 6.90, 7.91)	0.892
Week 8	24.9 (17.0)	24.9 (16.6)	0.03 (− 8.00, 8.06)	0.994
Week 12	22.8 (18.0)	24.5 (15.7)	− 1.68 (− 9.77, 6.40)	0.679
**SF12 Physical Score**				
Week 0	40.9 (9.0)	40.8 (8.9)	0.06 (− 4.22, 4.33)	0.979
Week 4	42.7 (10.8)	41.8 (7.5)	0.97 (− 3.51, 5.44)	0.668
Week 8	43.7 (10.8)	40.7 (9.1)	2.94 (− 1.84,7.72)	0.223
Week 12	44.3 (9.3)	42.3 (9.9)	1.9 (− 2.57, 6.55)	0.387
**SF12 Mental Score**				
Week 0	45.7 (12.2)	45.0 (10.6)	0.71 (− 4.70, 6.20)	0.796
Week 4	47.1 (10.9)	46.4 (10.9)	0.70 (− 4.50, 5.90)	0.788
Week 8	46.3 (11.8)	45.3 (9.9)	0.95 (− 4.30, 6.20)	0.716
Week 12	48.4 (10.7)	47.5 (10.5)	0.89 (− 4.20, 5.90)	0.726

### Numeric pain score

Both groups showed statistically and clinically significant differences in the NPS between the following time points: weeks 0 and 4, weeks 0 and 8, and weeks 0 and 12. In the placebo group, there was a 2.4-point improvement (*p* = 0.000) between weeks 0 and 4, a 2.6-point improvement (*p* = 0.000) between weeks 0 and 8, and a 3.6-point improvement (*p* = 0.000) between weeks 0 and 12. In the shockwave group, the improvements were a 1.9-point increase (*p* = 0.000) between weeks 0 and 4, a 2.1-point increase (*p* = 0.000) between weeks 0 and 8, and a 2.3-point increase (*p* = 0.000) between weeks 0 and 12 (see Table 4).

**Table 4:** Results of changes between weeks in NPS, PPT, NDI and SF12 scores within each treatment groups using paired t test

		**Placebo group (*n* = 36)**			**Shockwave group (*n* = 34)**		
**Measure**		Mean difference (SD)	(95% CI)	*p*-value	Mean Difference (SD)	(95% CI)	*p*-value
**NPS**	0–4 weeks	2.4 (0.50)	(1.4, 3.4)	0.000	1.9 (0.44)	(1.04, 2.8)	0.000
	0–8 weeks	2.6 (0.49)	(1.6, 3.6)	0.000	2.1 (0.42)	(1.3, 3.1)	0.000
	0–12 weeks	3.6 (0.49)	(2.6, 4.6)	0.000	2.3 (0.40)	(1.5, 3.1)	0.000
**PPT**	0–4 weeks	− 0.48 (0.21)	(− 0.93, − 0.04)	0.030	− 0.5 (0.17)	(− 0.94, − 0.23)	0.002
	0–8 weeks	− 0.51 (0.21)	(− 0.96, − 0.08)	0.020	− 0.5 (0.17)	(− 0.90, − 0.17)	0.005
	0–12 weeks	− 0.66 (0.25)	(− 1.17, − 0.15)	0.010	− 0.6 (0.17)	(− 1.04, − 0.32)	0.000
**NDI**	0–4 weeks	6.47 (2.6)	(1.15, 11.78)	0.010	5.56 (2.1)	(1.23, 9.90)	0.010
	0–8 weeks	6.13 (2.7)	(0.56, 11.72)	0.030	4.75 (2.6)	(− 0.56, 10.07)	0.070
	0–12 weeks	8.22 (2.9)	(2.15, 14.28)	0.000	5.12 (2.7)	(-.44, 10.69)	0.060
**SF12 Physical Score**	0–4 weeks	− 1.8 (1.1)	(− 4.24, 0.50)	0.110	− 0.9 (1.1)	(− 3.22, 1.29)	0.392
	0–8 weeks	− 2.8 (1.1)	(− 5.11, − 0.51)	0.010	0.07 (1.4)	(− 2.97, 3.12)	0.959
	0–12 weeks	− 3.4 (1.4)	(− 6.38, − 0.45)	0.020	− 1.4 (1.7)	(− 5.11, 2.15)	0.412
**SF12 Mental Score**	0–4 weeks	− 1.4 (6.3)	(− 3.55, 0.70)	0.183	− 1.42 (8.2)	(− 4.30, 1.45)	0.320
	0–8 weeks	− 0.6 (7.5)	(− 3.17, 1.91)	0.617	− 0.3 (10.4)	(− 4.05, 3.28)	0.832
	0–12 weeks	− 2.7 (8.8)	(− 5.74, 0.21)	0.06	− 2.5 (9.7)	(− 5.99,.83)	0.133

### Pain pressure threshold

Similarly, both groups demonstrated statistically and clinically significant differences in the PPT across weeks 0, 4, 8, and 12. In the placebo group, there was a − 0.48-point improvement (*p* = 0.03) between weeks 0 and 4, a − 0.51-point improvement (*p* = 0.02) between weeks 0 and 8, and a − 0.66-point improvement (*p* = 0.01) between weeks 0 and 12. For the shockwave group, the findings indicated a − 0.5-point improvement (*p* = 0.002) between weeks 0 and 4, a − 0.5-point improvement (*p* = 0.005) between weeks 0 and 8, and a − 0.6-point improvement (*p* = 0.000) between weeks 0 and 12 (see Table 4).

### Neck disability index

Both groups demonstrated statistically and clinically significant improvements in the NDI score between weeks 0 and 4. The placebo group showed a 6.47-point improvement (*p* = 0.010) between weeks 0 and 4, a 6.13-point improvement (*p* = 0.005) between weeks 0 and 8, and an 8.22-point improvement (*p* = 0.000) between weeks 0 and 12. In the shockwave group, there was a 5.56-point improvement (*p* = 0.010) from weeks 0 to 4. However, in the shockwave group, no significant differences were noted between weeks 0 and 8 (*p* = 0.070) or between weeks 0 and 12 (*p* = 0.060) (see Table 4).

### SF- 12 mental and physical scores

With respect to the SF- 12 scores (mental and physical), the shockwave group did not significantly differ between weeks 0 and 4, 0 and 8, or 0 and 12. In contrast, the placebo group demonstrated a statistically significant improvement in the SF physical score between weeks 0 and 8 (*p* = 0.01) and between weeks 0 and 12 (*p* = 0.02). However, there was no significant difference between weeks 0 and 4 (*p* = 0.110). Additionally, the placebo group showed no statistically significant differences in the SF mental score across weeks 0, 4, 8, and 12 (see Table 4).

The results of an independent t test between the placebo and shockwave groups revealed no significant differences in the NPS, PPT, NDI, or SF12 scores between the groups at weeks 0 and 4, 0 and 8, or 0 and 12 for these measures (*p* > 0.5). See Table 5.

**Table 5:** Results of changes between weeks in NPS, PPT, NDI and SF12 scores between the treatment groups using independent t-test

**Measure**		**Placebo group**	**Shockwave group**		
		Mean difference (SD)	Mean difference (SD)	95% CI of the difference	*p*-value
***NPS***	0–4 weeks	2.4 (0.50)	1.9 (0.44)	0.50 (− 0.87, 1.82)	0.484
	0–8 weeks	2.6 (0.49)	2.2 (0.42)	0.40 (− 0.89, 1.73)	0.525
	0–12 weeks	3.6 (0.49)	2.3 (0.40)	1.30 (0.017, 2.59)	0.047
***PPT***	0–4 weeks	− 0.48 (0.21)	− 0.5 (0.17)	0.10 (− 0.46, 0.66)	0.718
	0–8 weeks	− 0.51 (0.21)	− 0.5 (0.17)	0.02 (-.55, 0.58)	0.951
	0–12 weeks	− 0.66 (0.25)	− 0.6 (0.17)	0.02 (-.59, 0.64)	0.942
***NDI***	0–4 weeks	6.47 (2.6)	5.56 (2.1)	0.90 (− 5.87, 7.6)	0.791
	0–8 weeks	6.13 (2.7)	4.75 (2.6)	1.38 (− 6.2, 8.96)	0.718
	0–12 weeks	8.22 (2.9)	5.12 (2.7)	3.09 (− 5.01, 11.20)	0.449
***SF12 Physical Score***	0–4 weeks	− 1.8 (1.1)	− 0.9 (1.1)	− 0.91(− 4.1, 2.31)	0.5753
	0–8 weeks	− 2.8 (1.1)	0.07 (1.4)	− 2.9 (− 6.6, 0.84)	
	0–12 weeks	− 3.4 (1.4)	− 1.40 (1.7)	− 1.93 (− 6.51, 2.64)	0.4020
***SF12 Mental Score***	0–4 weeks	− 1.4 (6.3)	− 1.42 (8.2)	0.01 (− 3.48, 3.49)	0.9978
	0–8 weeks	− 0.6 (7.5)	− 0.30 (10.4)	− 0.25 (− 4.58, 4.09)	0.9103
	0–12 weeks	− 2.7 (8.8)	− 2.50 (9.7)	− 0.18 (− 4.62, 4.26)	0.9354

A one-layer multiple regression evaluating the impact of baseline characteristics on scores from weeks 0–4, 0–8, and 0–12 revealed that baseline variables were not statistically significant in predicting outcomes.

### Adverse events and dropouts

No dropouts were reported due to adverse effects of the therapies. Only two patients in the radial shockwave group experienced some temporary pain sensitivity as an adverse event, but neither of them dropped out. The number of dropouts due to loss to follow-up was comparable between the radial shockwave and placebo groups.

## Discussion

The results of this study indicate that there was a statistically and clinically significant improvement (*p* < 0.05) in both the NPS and PPT. This improvement was observed at the follow-up assessments conducted at 0–4 weeks, 0–8 weeks, and 0–12 weeks within the groups. Within the placebo group, there was a statistically and clinically significant difference in the NDI score at 0–4 weeks, 0–8 weeks, and 0–12 weeks. In the shockwave group, a statistically and clinically significant difference in the NDI score was found only at 0–4 weeks. Cultural differences may have influenced the outcomes of the study. In countries such as Saudi Arabia and other Asian and Middle Eastern nations, patients often expect a variety of treatment options, including modalities such as RSWT, and they believe that these approaches enhance pain relief. In contrast, individuals in Western countries tend to be more skeptical of such treatments, which may lead to a lower incidence of treatment bias [48]. Additionally, the therapeutic home exercises provided to both groups may have contributed to the positive outcomes observed in the shockwave and placebo groups.

Another plausible explanation for the similar outcomes between the groups in this trial could be the therapeutic effect of the energy flux density of both the shockwave 1.5 bar (0.068 mJ/mm^2^) and the placebo 0.3 bar (0.01 mJ/mm^2^). It is possible that the placebo group, which was exposed to an energy flux density of 0.3 bar (0.01 mJ/mm^2^) experienced some physiological effects similar to those of the RWST. In a related study that investigated the effects of extracorporeal shock wave therapy on the thigh muscles of healthy athletes, the group treated with an energy flux density of 0.03 mJ/mm2 presented improvements in muscle elasticity, tone, and recruitment compared with the control group [49]. The authors suggested that the biological effects of ESWT on the connective tissue of athletes could be attributed to increased blood flow, oxygenation, activation of metabolic processes, and a proliferative effect. In a study by Ji et al. [50] on the effectiveness of ESWT for MPS in the upper trapezius, the shockwave group was treated with an energy flux density of 1.24 bar (0.056 mJ/mm2), whereas the control group received 0.02 bar (0.001 mJ/mm2). According to Rompe et al. [25] the general ranges for ESHT energy levels are as follows: low (< 0.08 mJ/mm^2^), medium (0.08–0.28 mJ/mm^2^), and high (> 0.29–0.60 mJ/mm^2^). This suggests that the specific cut-offs for energy levels may vary across different studies. While there is no clear consensus on the minimum therapeutic dose of ESWT [51, 52], the appropriateness of the placebo level in our study was determined by prior research on ESWT [25, 52]. Future studies should further explore this aspect.

No statistically or clinically significant differences were observed between the placebo and shockwave groups across all outcomes at 4, 8, and 12 weeks. These findings are consistent with those of previous studies, including a meta-analysis by Jun et al. [22], which examined 11 randomized controlled trials and concluded that the RSWT was not significantly effective in reducing pain intensity or the pressure pain threshold (PPT) in patients with myofascial pain syndrome (MPS) in the neck and shoulder regions. Similarly, Yoo et al. [35], in a systematic review and meta-analysis of the effects of focused ESWT on MPS of the trapezius, reported no concrete evidence supporting the efficacy of this therapy for short-term relief of neck pain in patients with MPS. Moreover, In contrast, a prior study by Ji et al. [50] revealed that extracorporeal shock wave therapy was effective in reducing pain and the PPT in patients with MPS affecting the upper trapezius. However, a significant limitation of this study is its small sample size of only 20 participants and very short follow-up period of only 2 weeks. Additionally, while the study employed a placebo-controlled design, Ji et al. [50], did not clarify whether the participants were randomized via concealed allocation. To our knowledge, this is the first proper placebo-controlled randomized trial investigating the effectiveness of the RSWT in patients with MPS.

### Clinical implications

The results of this study on the RSWT align with those of previous studies, including the findings of Luan et al. [15]. In their study, Luan et al. [15] reported improvements in the visual analogue scale, pressure pain threshold (PPT), and neck disability index following an RSWT for pain in the upper trapezius, as assessed at the 3-month follow-up. Similarly, Manafnezhad et al. [36] reported comparable results just 3 weeks after the intervention. In recent years, clinicians have increasingly utilized SWT to treat musculoskeletal pain, particularly MPS in the upper back and trapezius areas [53]. This is particularly important given that musculoskeletal pain is a pressing global health concern that significantly burdens individuals and society. Based on these findings, which are consistent with those of previous studies [15, 36], the consideration of RSWT as a supplemental treatment for short-term benefits is warranted. The therapeutic home exercises given to both groups may have contributed to the positive outcomes in the shockwave and placebo groups.

### Strengths and limitations of the study

The trial had several strengths. It was prospective, registered, and employed a robust randomization process, ensuring that the two groups were similar at baseline. The concealed allocation not only prevented allocation bias but also minimized treatment bias since the physical therapists involved in the trial did not determine which group received treatment. The pragmatic nature of the trial reflected real-life clinical practices, thus enhancing the generalizability of the study. Additionally, this design meant that there were no extra costs involved. The trial utilized trained and skilled physical therapists for the use of radial shockwave therapy in treating patients with myofascial pain syndrome, which improved treatment fidelity.

This study has several limitations. Twenty-nine percent of the randomized participants were lost to follow-up at four weeks. Although this was not significantly different between the groups, we acknowledge that non-adherence can lead to unmeasured bias in intention-to-treat results [54, 55]. Future studies should consider the potential challenges of participants’ compliance with study protocols, including fixed religious calendars and school holiday periods. The effects of these areas have not been documented in research investigating the lack of participants’ compliance with study protocols.

## Conclusion

No significant differences were found between the placebo and shockwave groups at 4, 8, and 12 weeks. However, both groups showed statistically and clinically significant improvements in the NPS and PPT. Both groups showed improvements in the NPS and PPT scores; therefore, we recommend using radial RSWT as an adjunct to standard care, which includes therapeutic home exercises for individuals with MPS.

## Supplementary Information

Supplementary Material 1.

## Data Availability

The data presented in this study are available upon request from the corresponding author.

## Abbreviations

CONSORT: Consolidated standards of reporting trials
ESWT: Extracorporeal shockwave therapy
ITT: Intention-to-treat
KFSHRC: King faisal specialist hospital and research centre
MPS: Myofascial pain syndrome
NDI: Neck disability index
NICE: National Institute for Clinical Excellence
NPS: Numeric pain scale
PPT: Pressure‒pain threshold
PT: Physical therapist
RCT: Randomized controlled trial
RSWT: Radial shockwave therapy
SF- 12: Short-form health survey
SWT: Shockwave therapy
